# Invalidated and ‘salty’: an auto/biographical and theoretical review of the lived experiences of individuals with PoTS

**DOI:** 10.3389/fsoc.2024.1283695

**Published:** 2024-06-04

**Authors:** Harriet Marks

**Affiliations:** School of Society and Culture (Faculty of Arts, Humanities and Business), University of Plymouth, Plymouth, United Kingdom

**Keywords:** invisible illness, health interactions, PoTS, dysautonomia, lived experience, COVID-19, disability, auto/biography

## Abstract

Postural orthostatic Tachycardia Syndrome (PoTS), sometimes also written as ‘POTS’, is a form of dysautonomia (dysfunction of the autonomic nervous system) and orthostatic intolerance (which causes symptoms to be worsened when standing). This paper explores the extant literature on the lived experiences of those living with PoTS in relation to interactions between patients and healthcare providers as well as interactions at the level of the individual between PoTSies and those around them. My title contains the word ‘salty’ because it can be used to describe the feeling of being frustrated, while also reflecting a specific dietary change recommended to many (but not all) PoTS patients when they are told to consume additional sodium to minimise symptoms. COVID-19 is thought to have led to an increased prevalence of PoTS so this topic is particularly relevant to contemporary discussions and debates. In this sociological article, I refer not only to existing research on the lived experiences of having PoTS but also that of other chronic illnesses when relevant. The following themes are explored through auto/biographical and theoretical analysis: Undiagnosed and Invalidated; (In)Visible; Impacts of Diagnosis; Recovery and Expectations; Community. Reflecting auto/biographically, I have included analysis of interactions related to my lived experiences of presyncope, COVID-19 and dysautonomia, as I have been diagnosed with PoTS myself, which is thought to have been significantly exacerbated by the COVID-19 virus. This research is sociological, rather than medical or psychological, and conclusions are drawn about what is known so far about the lived experiences of living with PoTS, as well as discussion about what remains unknown, as there is currently a paucity of research on the lived experiences of individuals with PoTS and its comorbidities.

## Introduction

In this paper, I thematically, auto/biographically analysed the lived experiences of those with PoTS in relation to interactions between patients and healthcare providers as well as interactions at the level of the individual between PoTSies and those around them. I conducted the theoretical aspect of the review by exploring extant literature and secondary data on the ways in which interactions are experienced and perceived by individuals with PoTS and similar or related chronic (long-term) illnesses. ‘Understanding health, illness, disability, and medical interactions through a sociological framework is the basis of the sub-discipline of medical sociology’ (Frane, 2023: 10) and as Frye et al. (2022: 623) asserted, ‘the lack of qualitative methodology [in research on patients’ experiences of PoTS] is concerning, given qualitative data can improve our understanding of patient impairments, needs, and experiences, which may facilitate patient recommendations or services’. As Morgan (1998: 655) explained, auto/biography is ‘not simply a shorthand representation of autobiography and/or biography but also [a] recognition of the inter-dependence of the two own lives’ (in Letherby, 2022: 14). Sharing patient voices, including through auto/biographical research, may be vital in addressing, and working towards preventing, the social challenges of having disabilities/chronic illnesses such as PoTS so that in future, there can be more understanding from others. This introductory section of my paper summarises the findings of key texts in the field and the rationale behind my themes, as well as how and why I incorporated auto/biographical analysis.

Initially, my research aims were to understand the impacts of social interaction, condition invisibility and misdiagnosis/ delayed diagnosis on individuals with PoTS. While a small number of texts qualitatively explored the lived experiences of those with PoTS (such as Waterman et al., 2021; Frye et al., 2022; Knoop and Dunwoody, 2022), and Frane’s (2023) recent thesis explored the lived experiences of those with PoTS and Ehlers-Danlos Syndrome (EDS), I soon realised that there was/is a paucity of research in my chosen area. In these key texts, the authors analysed themes such as reduced functionality, invisibility, (in)validation, advocacy, condition management, adapting to cope, loss of control and agency, identity changes, mixed feelings, being expensive in many ways, gendered issues and the journey to diagnosis (Waterman et al., 2021; Frye et al., 2022; Knoop and Dunwoody, 2022; Frane, 2023). My paper’s final themes emerged through a combination of manually reviewing the interaction-related experiences of myself, other PoTSies in previous research and non-academic sources of patient stories such as some of those on the PoTS UK (a) website. I decided to structure my review somewhat chronologically, focusing on the lived experiences within different stages of a PoTS journey from being undiagnosed, to the impacts of diagnosis, to post-diagnosis life with PoTS and expectations for the future. In addition, I explored the varied visibility of PoTS (including examples of the illness being visible, partially visible and/or invisible) and (un)helpful experiences of community support groups. In my paper, I referred to wider sociological texts and theories such as Goffman’s work (Goffman, 1959, 1963) and Parson’s sick role paradigm (in Kelly and Millward, 2004: 6–7; Cheshire et al., 2021: 299) to contextualise discussions.

**Figure 1:**
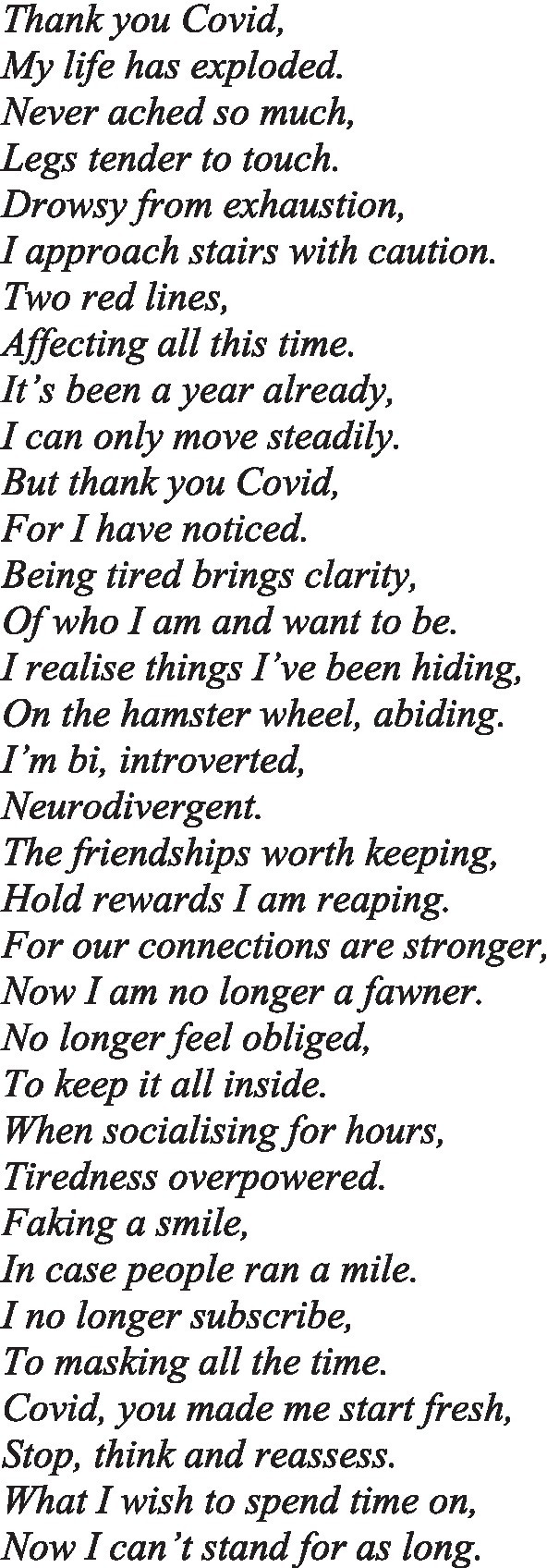
Thanks a lot, COVID.

**Figure 2:**
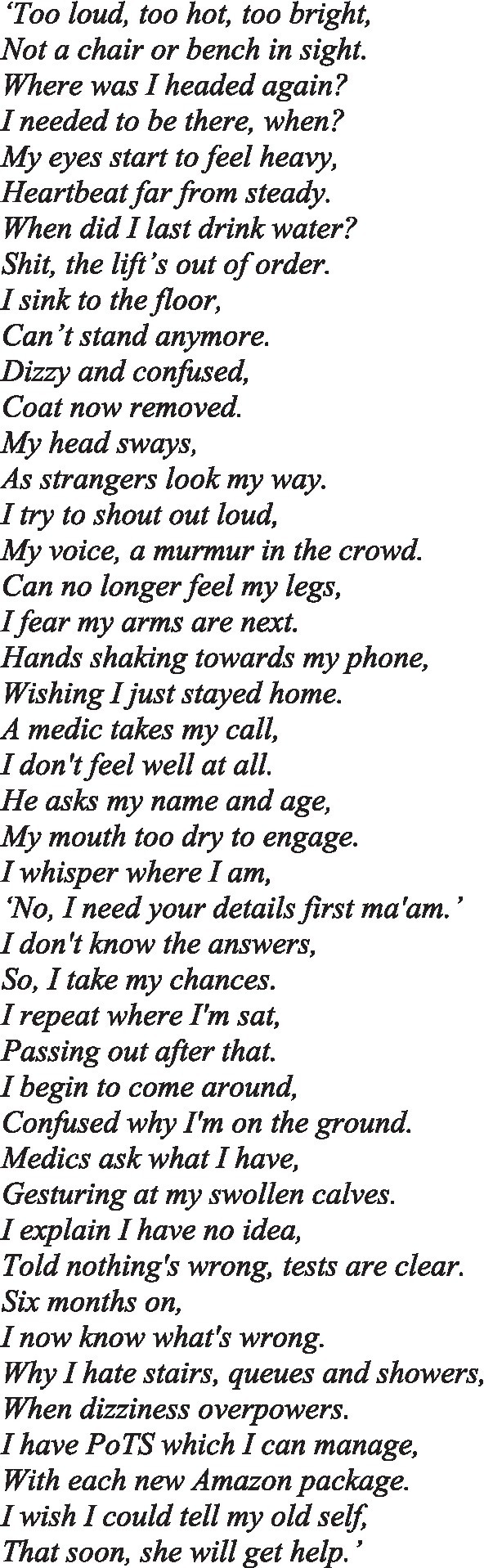
Dizzy and confused.

As Moore (2013: 201) explained, articles on illness stories have tended to be ‘constructed by a third party, the researcher, an individual looking at the story from the outside, understanding the story from their (perhaps removed) perspective and interpretative lens’. However, she used autoethnography to ‘try to offer a “true” picture’ of living with ulcerative colitis (Moore, 2013). I was inspired by her paper and the ways in which she analysed her experiences, endeavouring to write an article like this of my own. As Frane (2023: 11) explained in her autoethnographic thesis, ‘my own identity and lived experiences have contributed to the framing of the research aims I sought to explore, as well as influencing the lens through which I conducted my research’. While these two papers used autoethnography, my article utilises auto/biography. Despite some scholars criticising auto/biography, Letherby (2022: 14) posited that ‘all research and (scholarly) writing is in some ways auto/biographical, involving intersections of the lives of those who write and those who are written about’ and ‘all texts bear traces of the author’. I incorporated my lived experiences through various stages of my journey with dysautonomia, presyncope and COVID-19 as well as the social, medical and emotional impacts of receiving a diagnosis of PoTS. I chose to share my story because I wanted to help others and aid healthcare professionals (HCPs) and able-bodied members of the public in gaining understanding, in depth, of what it is like to be chronically ill and how interactions can be experienced by someone with a diagnosis of PoTS. I wrote two poems about my lived experiences shortly after my PoTS diagnosis, which I have included to help convey my emotions (Figures 1, 2). Although using ‘I’ can still present challenges to more traditional academic writing, like Davies (2012: 7), I hope that ‘by writing in the first person, I have produced a more effective piece of research and writing’, which can help others.

The remainder of my paper is split into three sections: ‘Background and Context’, ‘Theoretical and Auto/Biographical Review’ and ‘Discussion and Conclusion’. In the former, I began by introducing my story, before giving an overview of what PoTS is and how it can present. In the next section, I explored the following themes subsequent to conducting an auto/biographical and theoretical analysis, combining the lived experiences of myself and PoTSies in previous research: Undiagnosed and Invalidated; (In)Visible; Impacts of Diagnosis; Recovery and Expectations; Community. Lastly, in the ‘Discussion and Conclusion’, I summarised this review’s findings, limitations and proposed areas for future research.

## Background and context

When I first caught COVID-19 in January 2022, I felt particularly dizzy, fatigued, confused and sensitive to heat. I struggled to stand up for long or to stay awake. Before the virus, I used music when I felt tired and needed to feel more alert so a couple of weeks after my positive PCR test, I tried putting on a song and dancing to it. Usually, I would dance for 10–15 min and then feel ready for the day or task ahead of me but I swiftly realised I felt too weak and unbalanced to stay on my feet. I realised I had been dancing for less than a minute and assured myself that although I was in a lot of pain and felt upset by the disruption to my plans and my life, it was a temporary bug, I was young and it would pass. As the months went on, things were improving at a shockingly slow pace. Before, I loved cooking curry, stir-fries and pasta dishes but the heat from the kitchen made me feel like I was going to faint so I mostly stuck to microwaving soup. I could go to work but I had to stop and sit several times on my way there, as the short distance I could previously walk with ease was still challenging for me to navigate 5 months after infection. In Moore’s (2013: 204) paper, she described her chronic illness as ‘The Beast’, stating, ‘I feel The Beast within me beginning to tighten its grip. […] I want to just rip it out of me. I want to be able to run around, train with [her football] team. I want my body back, back to normality’. This resonated with me as I saw my post-Covid symptoms as a destructive and disruptive force and wanted to feel how I did before.

Almost 7 months after my first bout of Covid, I found out that I had caught it again. This was distressing news, as I felt emotional and scared about how it would impact my health. My friend texted me to ask if I felt better this time and I replied, ‘No.’ before explaining, ‘I’m so achy, dizzy and fatigued. I’ve defo [meaning definitely] felt I’ve had [L]ong Covid anyway and […] I’m completely out of breath from going up the stairs like I was last time’. This time, my symptoms became more severe, which was not helped by it now being the summer because ‘heat exposure’ (Bryarly et al., 2019: 1222) and ‘environmental heat’ (Bryarly et al., 2019: 1223) can exacerbate symptoms of orthostatic intolerance (OI). This time also held more social offers from friends, which I was almost entirely declining. This included saying goodbye to a friend who was moving away, going to the beach, the cinema, shopping, for meals, a trip to another city and anything that involved alcohol, as alcohol caused me to flare-up and I now know that it can exacerbate OI symptoms (Bryarly et al., 2019: 1222). Moore (2013: 204) reflected on being asked to play in a football match, stating, ‘“YEEEEESSS” screams my heart’ but that she knows her body’s answer would be different; ‘Which one should I listen to?’ (Moore, 2013). This encapsulated how conflicted I felt, as I longed to do everything that my body was unable to do. I felt deflated when declining opportunities but when I accepted things I should not have done, my mind was writing a cheque that my body could not cash. The summer heat made me feel confused and dizzy and being put on medical waiting lists made me feel impatient. I just wanted to be able to live my life again and to be ‘able’, full stop.

While large questionnaire-based studies ‘have detailed the symptoms people with PoTS experience, very little has been described about the intricacies and ongoing impact of these on daily life’ (Waterman et al., 2021: 185). It took approximately 17 months from my first infection of COVID-19 to my diagnosis of PoTS and much of that time was rife with frustrating interactions with HCPs, leading to invalidation and fear. It is believed by my HCPs and I that I likely had dysautonomia before catching the virus so COVID-19 exacerbated, rather than caused, my symptoms. Postural orthostatic Tachycardia Syndrome (PoTS), also written as ‘POTS’, is ‘a type of autonomic dysfunction often characterised by orthostatic intolerance, dizziness, fatigue, syncopal events, nausea, dyspnea, and excessive postural tachycardia’ (Frye et al., 2022: 1). It mostly affects young women (Ormiston et al., 2022: 1881; Fedorowski, 2019: 352; Dipaola et al., 2020: 2) and is the most prevalent form of OI and dysautonomia (Ormiston et al., 2022: 1881). Symptoms and severity vary and there is a paucity of research on the lived experience of individuals with PoTS, despite this chronic illness having an ‘enormous impact on patients’ quality of life’ (Fedorowski, 2019: 353). I hope that this paper conveys the impacts of health and illness interactions on the lived experiences of individuals with PoTS, who have an often-invisible chronic illness, as well as helping those who have, or will later develop, the condition.

## Theoretical and auto/biographical review

### Undiagnosed and invalidated

Dipaola et al. (2020: 2) stated that ‘patients are often underdiagnosed or misdiagnosed and are evaluated on average by seven doctors before receiving a POTS diagnosis’ and ‘the median diagnostic delay is 24 months, with peaks reaching 10 years’, although in Courtney and Lesley’s stories on PoTS UK (a), they described struggling for 17 years and over 20 years before their diagnoses (respectively). In Knoop and Dunwoody’s (2022: 1631) study, ‘the importance of being heard and believed was a central aspect of living with POTS’ and ‘this was complicated by misdiagnosis, which led to anger, feelings of being discredited and “fighting” for a diagnosis’. Similarly, Frye et al. (2022: 5) found a common theme in their research was ‘medical professionals not believing’ adolescents with PoTS with one participant stating, ‘I just felt like [my doctor], had his mind set already’. ‘Feelings of dismissal and invalidation were reported to negatively impact the family and the adolescent’s emotional well-being’ (Frye et al., 2022: 6). One parent stated that in the car after specialist doctors’ appointments, her daughter would ‘break down just in hysterics and […] be crying and sobbing’, ‘saying what’s wrong with me, why do they keep telling me there is nothing wrong with me’ (Frye et al., 2022). This resonated with my experience of trying to work out the cause of my symptoms, as the phase in which I was undiagnosed—particularly before I had heard of PoTS/dysautonomia and started suspecting I may have it—was the most stressful in my health journey to date. When I told one doctor how much I was struggling since catching COVID-19 twice and having a severe flare-up from a flu jab, he replied that many people were tired since being infected by the virus, including him, and that he just went to bed earlier. I found this very rude and invalidating because rest did not refresh me as it did him and if an earlier bedtime was all I needed, I would not have been having the appointment with him in the first place.

While there is a paucity of academic research on the lived experiences of the journey from being undiagnosed to receiving a PoTS diagnosis, Spillmann et al. (2017: 1) found that individuals with undiagnosed illnesses expressed frustration at being undiagnosed and adults felt ‘they had to provide validation of their symptoms to providers, given the lack of objective findings’. In Dóczy’s (2022) article, she reflected on having an undiagnosed illness, stating that ‘the hardest part for me is waking up to hopelessness every day, starting each new day with pain – because the pain is all day, every day’. She listed activities that she used to do before, stating ‘now everything is lost’ and ‘without health, everything feels impossible to do’. Lacking and seeking a diagnosis can cause frequent appointments and tests and personal, familial, emotional and financial stresses (Spillmann et al., 2017: 2). BJGP Life (2023) explored experiences of having an undiagnosed illness, stating, ‘Susan has been examined by 20 doctors’ leading to ‘only a long list of rule-outs’. ‘The function of a diagnosis is more than to guide treatment planning. It often provides emotional relief for patients, even if the diagnosis is dire’, with Susan explaining, ‘I keep hoping that some doctor will tell me exactly what this ‘skin issue’ is, even if there’s no cure’ (BJGP Life, 2023). When seeking a diagnosis, some HCPs may be more validating than others, for example, one specialist’s delivery was particularly understanding and considerate towards me as she phrased the lack of answers or diagnoses as a negative, stating that she was sorry to inform me that she could not be of more help. However, most HCPs that I interacted with presented the news as positive, almost using it to imply that I had nothing wrong with me, which was frustrating as I felt no closer to knowing what I had or what I could do to treat it. I was once told by an HCP, ‘you feel dizzy because you are skinny’ but since catching COVID-19, I have been told multiple times that my dizziness must be the result of me being overweight. Both felt/feel frustrating, hurtful and invalidating because since puberty, I have struggled with presyncope and fatigue regardless of how much or little I weigh and when HCPs focused on my weight, it made me feel concerned that they were not seriously searching for the cause of my symptoms.

Some of my post-Covid symptoms were new to me but over time, I realised that the vast majority were not. Realising that I may have had a condition that worsened from the virus, rather than just taking a while to recover from infection, was somewhat validating but also nerve-wracking as I worried about whether the time delay between initial symptom onset (believed to be around puberty) and my viral infection (in my early 20s) could mean that I had worse prospects for the future than if whatever was wrong had been identified sooner. I had a pelvic and abdominal ultrasound to find out if there were any signs of endometriosis (or a cyst) and when the HCP performing this asked me what else I had on for the day, I replied, ‘I’m having this because my main symptom is fatigue, I’m not very well at the moment so I will need to go to bed after all the walking to get in here’. ‘Ooooh lucky you, I’m soooo jealous – I’d love a day off’, he replied animatedly. I felt frustrated and wanted to cry but felt that because he was holding the medical wand that was inside of me and my legs were in stirrups, this was probably not an appropriate time to express myself. Although he probably had good intentions, I did not want to have to go back to bed and felt that he would not be jealous of what was happening to me if he truly understood how draining and challenging it was. Spillmann et al. (2017: 1-2) explored three types of illness narratives identified by Frank’s past research: ‘restitution (expectation of recovery), chaos (suffering and loss), and quest (unexpected positive effect from illness)’. ‘Living with an undiagnosed condition prevents the probands from being able to transition out of chaos to quest and the very nature of chaos prevents them from being able to clearly communicate their illness story’ (Spillmann et al., 2017: 9). Similarly, in Frane’s (2023: 72) thesis, ‘the conclusion to which most [participants’] illness narratives built tended to be that of the point of diagnosis’.

A survey found that in the almost 4 years on average from symptom presentation to obtaining a PoTS diagnosis, psychiatric mislabelling was ‘common’, as 48% of respondents were advised that symptoms were ‘due to a psychological or psychiatric disorder, such as anxiety, panic disorder, depression, or hypochondriasis’ (Kavi et al., 2016). On PoTS UK (a), Chloe stated that her ‘PoTS story’ began in 2020 after she tested positive for COVID-19 and became ‘very unwell with Long Covid and pleurisy’, which caused her heart to beat rapidly ‘but this was just put down to anxiety’. In May 2023, she began experiencing dizziness and a fast heart rate when she changed position and ‘after endless doctor’s appointments and monitoring of my heart I was eventually referred to a PoTS specialist [..] in August 2023’ (PoTS UK, a). ‘Although anxiety is […] commonly described in POTS, the excessive tachycardia is not simply a physiological manifestation of anxiety’ (Bryarly et al., 2019: 1216) and misdiagnoses of panic disorder or other psychiatric conditions (Waterman et al., 2021: 185) can prolong suffering. In one of my pre-diagnostic experiences, I went to my GP surgery to pick up a prescription and while I was there, I had my heart rate tested. During this, the HCP posited, ‘umm you have heart problem, yes?’, before dropping her pen and paper on the floor and seeming startled. She went to leave the room, leaving me attached to the machine still. ‘Is everything okay?’ I laughed because I thought it all seemed worse than it was. ‘No, very bad, very very very bad, heart is. very bad’, she replied, as she left the room hurriedly. When she returned, she informed me that I needed to see the urgent care team so I felt confused and numb.

The team were supportive and kind but it felt embarrassing having to take my T-shirt and bra off for the tests. Then, I sat with a medical professional to hear my results. Everything looked normal structurally with my heart (and my lungs which had also been tested) but my symptoms were apparently concerning. This was a relief but also stressful to hear. He said that I would need a 24-h ECG, a urine test and then his voice trailed off as he saw something else on his screen. I wondered which result he was looking at and started to feel worried by this reaction. ‘Oh. You picked up anxiety and depression medication today so nevermind’. I did not know what to reply. I explained that these had been prescribed to me to potentially help with my physical symptoms, particularly as low dose anti-depressants can help with gastrointestinal (GI) issues, and that I had not tried them yet. This was the truth but he looked at me as if it was not. He said that we were finished and I could go home and I felt lost and dazed about how anticlimactically such an unexpectedly stressful trip had ended. ‘Overall, the burden of living with an undiagnosed condition is high, with suffering, frustration and uncertainty’ (Spillmann et al., 2017: 8). For me, there were periods of anticipatory stress before a test, uncertainty and nerves when awaiting the results, followed by a recurrent and deflating anticlimax. While I was relieved at times not to have worse news and did eventually get the tests done that he had suggested and then un-suggested, I wanted an answer. Frustratingly, I also experienced my results being lost/misplaced before I received them multiple times (causing tests to have to be repeated), as well as referrals that my GP sent off (such as to neurology for my headaches and migraines) being rejected by the specialist departments. In Frane’s (2023: 69) thesis, participants with ‘a shorter diagnostic period’ or those who ‘struggled less with obtaining answers regarding their symptoms’ were diagnosed by private health professionals rather than the NHS and all but one of my appointments have been through the NHS, which may have affected my experiences.

Ormiston et al. (2022: 1881) stated that recent studies ‘have described patients recovering from COVID-19 as presenting with significant and debilitating POTS and POTS-like symptoms, suggesting that COVID-19 is yet another viral infection that can trigger POTS and that POTS is a distinct phenotype of long COVID’. In Au et al.’s (2022: 7) paper on Long Covid and medical gaslighting, one respondent wrote, ‘A nightmare. Gaslighting and denial and doubt. Dismissal. Western medicine has absolutely failed us’ (Au et al., 2022: 6) and a respondent with medical credentials recalled ‘being dismissed by their own colleagues’, as they explained, ‘I sought treatment at the healthcare system where I worked. I was treated like an anxious child. Nobody listens… Despite concrete evidence that something was wrong with me’ such as results of a heart monitor (Au et al., 2022). The paper stated, ‘while the majority of our [...] respondents were able to obtain a test, 86 (26%) [...] were unable to confirm their initial infection’. A long hauler explained, ‘in the beginning, it was terrifying. No one believed or understood that covid lasted longer than 2 weeks and it wasn’t a life-or-death thing. At the most terrifying point of my life I had to fight not just to live but for people to believe that my illness existed let alone to get help’ (Au et al., 2022). Interestingly, ‘the post-acute sequelae of COVID-19 is being diagnosed not only in those who developed severe acute COVID-19, but also in infected individuals who had mild and even asymptomatic cases’ (Plaut, 2023: 1). ‘There is a need for widespread education of health professionals about PoTS to avoid misdiagnosis and to facilitate timely diagnosis’ (Kavi et al., 2016). In Lesley’s story on PoTS UK (a), she reflected that upon receiving her diagnosis, ‘initially, I was so relieved that I wasn’t going crazy, then I realised that not only does my future look different to what I expected, my past also could have as well; if only I had been listened to, believed, and diagnosed sooner’. Hopefully, the increased prevalence of PoTS and PoTS-like conditions since the pandemic will increase research into, and awareness of, dysautonomia so that the time PoTSies go undiagnosed and invalidated is reduced/avoided.

### (In)visible

‘One of the challenges with POTS spanning diagnosis, treatment, and QoL [meaning quality of life] is the invisibility of this condition’ (Frye et al., 2022: 5). Kessler (2022: 57-59) analysed Cleasby’s story of being judged for using a disabled toilet despite having GI conditions and an ostomy, which ‘exposes the challenges of both living with an invisible disease and facing stigma’, through an ‘onlooking woman’s visual practices (staring) and verbal practices’ (tutting). A PoTSie from Waterman et al.’s (2021, 189) study stated, ‘I want people to understand that even though I look well...I really really am not feeling well.’ In Sophie’s story on PoTS UK (a), she reflected, ‘when people look at me, they see a normal, smiling teenager. [...] On the inside, things are very, very different’ and Chloe stated, ‘lots of people think there’s actually nothing wrong with me as I’m just a smiley, bubbly character. The hardest part about PoTS is not being able to do the things I used to do’ with walking up the stairs or getting out of bed being ‘so hard when your feel like the whole room is spinning constantly’. Kessler (2022, x-xi) stated that illnesses related to the digestive system are ‘an ideal case for rhetorically theorising stigma because these conditions sit on the edge’ of the boundary between being ‘invisible, until they become visible (visually, auditorily, or olfactorily)’. Similarly, my syncope and presyncope can sit on the edge of (in)visibility, as people may not realise that I feel like I am going to faint, cannot concentrate and am experiencing visual and auditory disturbances (due to presyncope) but do notice when I experience conscious blackout episodes, need to lay on the floor or start swaying.

One parent in Frye et al.’s (2022: 5) study on the lived experiences of adolescents with PoTS and their parents felt that children with invisible disabilities such as PoTS ‘look normal’ on the outside ‘but on the inside, they are struggling to get up every day and people do not take seriously what they are really going through’. The parent stated that she feels her children ‘go through more than the average adult does by the time they are 90 [years old]’ (Frye et al., 2022). In Poku and Pilnick’s (2022: 1036) study of the lived experiences of children and young people with sickle cell disease (SCD), one participant stated that saying she is tired after doing ‘something small’ makes people think ‘you do not want to do it, or you are lazy. They make it sound like you have decided to be tired’ and ‘they do not believe you because how can you be tired when you have almost done nothing and when you are not disabled or an older person who is weak and frail’. Another stated that tiredness makes him ‘feel like an old man’ and ‘it makes me feel bad because they tease me that I’m weak, girly, and there’s no fun in playing with me because I’m always complaining that I’m tired’ (Poku and Pilnick, 2022). These excerpts indicate that ‘reduced functionality and physicality are perceived as synonymous with old age and physical disability’ (Poku and Pilnick, 2022). Age may also impact PoTSies interactions with others, as it can be presumed that they look too young to be struggling in the ways that they claim to be. As a woman diagnosed with PoTS in my early 20s, I find it particularly invalidating when people tell me that I will know real muscle aches or tiredness when I am older or that I will know real hot flushes when I hit the menopause, as I already experience these symptoms severely now.

For individuals who develop PoTS, ‘the most common demographic is young, previously active women, and frequently, there is an identifiable event (such as illness, pregnancy, or surgery) that precedes symptom onset and precipitates withdrawal of activity or frank bedrest’ (Bryarly et al., 2019: 1225). Miles (1991: 88) argued that ‘problems of legitimacy and credibility loom larger in the illness experience of women than in that of men’, as well as asserting that ‘the widely-held stereotyped picture of woman as complaining, weak and inclined to magnify problems leads to general scepticism about the extent and severity of women’s symptoms. Much less are the symptoms of men called into question (Nathanson, 1975).’ A parent of an adolescent with PoTS in Frye et al.’s (2022: 6) study stated that her daughter’s school principal said to her, ‘I know you are playing everybody, but you cannot play me, I know there’s nothing wrong with you’. Because PoTS predominantly affects those assigned female at birth (AFABs), discussions around invalidation, invisibility and questioned credibility should be contextualised within the wider debate around whether AFABs’ health issues are taken as seriously as those assigned male at birth’s (AMABs’). As a woman and AFAB, I do not know the extent to which my gender or sex have contributed to my pre- and post-diagnosis experiences, however, I experience increased severity of symptoms preceding my period. ‘Women with POTS often report fluctuations in the severity of POTS symptoms throughout their menstrual cycle, with worsening during either the pre-menstrual or early follicular phase’ (Bryarly et al., 2019: 1212). However, these worsened symptoms rarely affect the visibility of my suffering, instead adding another invisible, often-invalidated struggle.

In Poku and Pilnick’s (2022: 1037) study, participants described the extent to which they felt pressured to meet masculine and feminine ideals. One male stated, ‘when I think of a man, what comes to mind is fitness, strength and energy levels’ (Poku and Pilnick, 2022), while another pursued ‘an unconventional biography informed by his own capabilities [such as drawing] rather than the conventional biographies of adolescence and masculinity’ (Poku and Pilnick, 2022: 1041). He said, ‘I do not see myself as weak because sometimes I do things my friends cannot do [...] Those who say I’m weak do not know me very well [...] [and] I do not think being strong is all about running or playing for long’ (Poku and Pilnick, 2022). Sometimes, I wonder whether I would receive as much help with carrying things in public places if I were not a young woman, for example, if I were a young man who was socially assumed to be strong. On PoTS UK (a), 38-year-old James described catching ‘Glandular Fever (EBV)’ and ‘within a year I could not fight the fatigue, insomnia and daily migraines any longer and had to stop working’. He explained, ‘they [HCPs] wrote me off as being depressed and having ME/CFS [myalgic encephalomyelitis/chronic fatigue syndrome]. I never really had the symptoms of depression, and I certainly did not meet the criteria for ME/CFS’, as well as stating that it was frustrating that his GP notes had said that he had a high heart rate multiple times, which they ‘put [...] down to “anxiety”’ despite him having tonsilitis or a headache and never complaining ‘about anxiety or anxiety symptoms’ (PoTS UK, a). 23-year-old Conor described having PoTS, Inappropriate Sinus Tachycardia (IST), Orthostatic Hypotension (OH), Neurocardiogenic Syncope (NCS) and OI (his ‘primary forms’ of dysautonomia), along with ‘chronic fatigue’, ‘digestive issues’ and mast cell activation syndrome (‘MCAS’), all stemming from hypermobile Ehlers-Danlos Syndrome (hEDS; PoTS UK, a). He stated, ‘I think it’s extremely important to share my story as a man with PoTS as it can be overlooked as a ‘woman only illness’, as I was once told by an incompetent doctor’ (PoTS UK, a). Thus, getting a PoTS diagnosis can also be subject to challenging, invalidating interactions for men/AMABs, not just women/AFABs.

After receiving a flu jab roughly 4 months after my second bout of Covid, I entered into a long, horrible flare-up and subsequent increased experience of syncope and migraine. Due to this, I sent my friend a voicenote saying that I was considering buying a walking stick but was unsure what people might think. I worried that others would ask why I was using it and thought I would have to admit that nothing had been found to be wrong with me that would justify its use. I knew it would help me to feel steadier, which would improve my QoL as I would be able to walk longer distances and have more independence but I doubted whether I was being overdramatic for wanting one. Stacey (a), a wheelchair user with moderate ME/CFS stated that she was ‘put off using a wheelchair for a long time’, as she ‘thought that they were only ‘allowed’ in certain cases and for certain people, and that [she] wasn’t one of them’. However, ‘my wheelchair allows me to do more of the things I love, with less detriment to my health’ as and when she chooses to use it (Stacey, a). The first time that I used my mobility aid, I felt amazing as it helped me to go into the optician’s without needing to hold onto anyone else and enabled me to feel secure and confident in the midst of dizziness and weakness. A theme identified in Waterman et al.’s (2021) study was ‘loss of control and lack of agency over body’ and when referring to those with rheumatoid arthritis, Nettleton (2013: 65) stated that individuals can become ‘dependent upon others’ and in ‘a culture which emphasizes independence and self-reliance, [this] can be threatening to the sufferer’s self-esteem’. It can ‘make social interactions, which in our society are for the most part based […] on reciprocity, particularly precarious’ (Nettleton, 2013). This resonated with me as it knocked my confidence having to depend on others when lacking agency over my body and I did not want to see my friends until I knew that if a dizzy spell occurred, I would be able to walk without linking arms or holding hands with them.

When I used my walking stick to go to work and occasionally to cafés or supermarkets, I felt thrilled that I had bought it but wished I had gotten it sooner. It would have made a huge difference to me to have bought this before my flu jab-induced flare-up, particularly when I went to my friend’s graduation a couple months before and stood for a long period of time feeling dizzy. Friends who I saw or spoke to frequently were unsurprised to see my walking stick and were happy for me that it had made such an improvement but some friends who I do not see often seemed to feel confused and/or perceive this negatively. Miles (1991: 87-88) stated that in cases of ‘episodic illness, psychiatric disorder and diseases which develop slowly and are not always apparent, it is the legitimacy of the condition itself’ that may need to be re-affirmed sometimes. When I told a friend I had seen at the graduation that I now had some limitations on what I could do when we met up, she kept saying that she was confused, including by my use of the word ‘disabled’ to describe myself. She remembered my symptoms but did not understand that this may cause me limitations sometimes and said, ‘I did not know you would say you were... *disabled?*’ The ‘invisible nature of the condition’ may not match up ‘with people’s expectations of someone with a disability’, with one PoTSie saying that they have been glared at for putting up their blue badge and asked if it is for them (Waterman et al., 2021: 189). For me, having PoTS is disabling due to its impacts such as me needing to sit down more often than able-bodied people. It was disabling before my walking stick and compact stool made it more visible and, in some ways, it was more disabling before I looked disabled.

This term ‘disabled’ can be viewed through the medical or social paradigms, with the former suggesting that people are disabled by their impairment(s)/difference(s). The latter, however, posits that individuals are disabled by barriers in society, not by their impairment(s) or difference(s; Oliver, 2013: 1024; SCOPE, n.d.; SENSE, n.d.); Societal barriers can be physical (such as a building not having a disabled toilet) or caused by people’s attitudes/ignorance (Oliver, 2013; SCOPE, n.d.; SENSE, n.d.). In Frye et al.’s (2022) study, one of the identified themes was ‘negative changes in functioning’ and Knoop and Dunwoody’s (2022: 1633) participants ‘not only coped with debilitating symptoms, but also the grief of lost friendships, previously enjoyed activities and to a certain extent, loss of independence’. Rich et al.’s (2020: 6) study identified ‘challenges with participation in functional daily activities, such as self-care, showering, cooking, shopping, spiritual activities, and doctor appointments’, which encapsulated ‘difficulties which were not directly linked to one symptom, but overall lead to additional challenges due to a lack of participation’. ‘Socializing with friends or family was interrupted due to routinely cancelling plans, which often led to withdrawal from future plans’ and ‘others reported a lack of energy to engage, inability to participate in activities, and fears surrounding being out in public due to safety concerns’ (Rich et al., 2020). I describe myself through medical terminology, my diagnosis was important to me and I see my impairment/chronic illness as somewhat disabling through the medical lens, however, I also feel that the extent to which I am (un)able to participate in activities/society is partially dependent on factors associated with the social model such as understanding from others and receiving help if I am unable to carry heavy items. Having the right accommodations such as being able to wear sunglasses indoors, refill my drink more often than other people and move my legs around to improve circulation make a huge difference to the extent to which my dysautonomia is disabling and to my confidence that I can retain control over my symptoms/body to participate socially. When I cannot control my environment, for example if all available lifts are out of order (which happens far more often than I realised before I was dependent on them), I am more risk-averse, fearful and less likely to participate in activities.

In Kavi et al.’s (2016) research on the experiences of individuals with PoTS, 23% were wheelchair users, 37% were unable to work, 5% were bedbound and 7% were mobile without restriction. I would describe myself as having ‘mobility issues’ as I can stand and walk but not for long without taking breaks. It takes me more energy than it would for an able-bodied person, as well as causing me to be more symptomatic and require longer to rest afterwards. Stacey (a) reflected that ‘because of the way mobility aids are typically portrayed in the media, I naively assumed they should only ever be a last resort, a worst-case scenario, viewed as an absolute tragedy’ but that actually, ‘using a wheelchair has given me my life back’. Ambulatory wheelchair users are people who use a wheelchair sometimes, rather than always, as despite possibly having some ability to stand, walk or move their legs, remaining seated may help them to avoid/reduce fainting, seizures, pain and/or over-exertion (Stacey, a). Stacey (b) discussed experiencing post-exertional malaise (PEM) in which symptoms flare-up after mental or physical activity, reflecting, ‘at my worst, it would only take accidentally standing for a couple of minutes longer than I should have done or walk[ing] a couple of metres further than my baseline, and I would go through hell for days afterwards [...] I’d be hollow with exhaustion’. However, her wheelchair has helped her to experience PEM much less, as ‘I began to feel more…okay. Not necessarily stronger, but much less breakable’ (Stacey, b). Despite advantages such as this, ambulatory wheelchair users, such as those with PoTS, may face abuse sometimes, as others see them standing up from their wheelchairs or moving their legs and presume that they do not actually need to be using one.

In Rich et al.’s (2020: 7) study, some participants ‘reported apprehension in leaving home without a wheelchair or other adaptive tool, fearing an episode of syncope or pre-syncope’. Individuals with visible illnesses may have less autonomy about disclosure than those with entirely or partially invisible disabilities (Joachim and Acorn, 2001: 245). Middleton (2023) explained that masking autism or other forms of neurodivergence can be exhausting and challenging but is also a privilege as those with high support needs, who are non-verbal/non-speaking and/or have comorbid (co-occurring) learning disabilities may not have the option to hide their traits to help keep themselves safe or prevent ostracisation. While I struggle to decide whether to wear my medical identification cards for PoTS and migraine on my lanyard and deliberate over the extent to which I want to disclose my health differences, it is a privilege that I can often choose whether to make my disabilities visible. For PoTSies who use wheelchairs, their disability’s visibility can prove challenging socially. Joachim and Acorn (2001: 244) state that ‘those without stigma generalise from a particular disability to a variety of disabilities or imperfections (Strauss et al., 1984)’, which may explain ‘why some people talk to a person in a wheelchair as if the afflicted cannot comprehend, shout at the blind, or speak to a companion of a disabled person rather than to the person with the disability’.

Cognitive challenges, also known as ‘brain fog, mental fog, mental clouding, or mental fatigue’ (Raj et al., 2018: 46), can also prove to be an invisible but debilitating aspect of having PoTS. Ross et al. (2013) found that over 95% of PoTS patients self-reported cognitive impairment (cited in Raj et al., 2018: 46; Rich et al., 2020: 2) and previous studies cited by Raj et al. (2018) suggested that depression and anxiety related to having a chronic’s illness can negatively impact cognition. In Rich et al.’s (2020: 7) research, ‘cognitive limitations identified by participants included issues with concentration, word-finding, focus and memory’ and nearly 20% of participants believed ‘these limitations impacted participation in daily life including decreased concentration and mental fatigue while driving, working, and during social activities’. I struggle with oscillating mental energy, as I can think clearly sometimes but this is dependent on my postural position, management of hydration (through consuming electrolytes), raising my legs, reducing sensory input and resting appropriately. When experiencing PEM, migraines or standing for too long, I find talking challenging so I am particularly cautious about trying to manage relevant factors including my energy before activities so that I can avoid running out of cognitive energy during them. I think most people would be surprised by the extensive effort I put in to prepare for activities and avoid cognitive issues, only seeing the version of me who can converse and present well, rather than all the work I have done backstage (Goffman, 1959) to achieve this.

However, some people with PoTS experience brain fog constantly or at least more independently of other factors than I do. For example, Raj et al. (2018: 46) stated that with PoTS, ‘importantly, this cognitive dysfunction can occur even while lying down or seated, limiting ability to engage in work and educational activities’. An adolescent with PoTS in Frye et al.’s (2022: 626) study described struggling with schoolwork due to brain fog and feeling ‘so dumb’, while a PoTSie participant in Rich et al.’s (2020: 7) study reflected, ‘my memory is horrid which also affects my social life. It’s hard to explain to people you have been around for years that you cannot remember their names or children’s names, and even complete conversations you have had with them’. The extent to which those around PoTSies are understanding about their cognitive challenges may impact their lived experiences, inclusion in social events, wellbeing and identity. Frye and Greenberg (2024: 3) referred to Frye et al.’s (2022) study, stating, ‘one participant reported concerns about independently cooking due to difficulties of brain fog and forgetfulness, that could lead to food or an oven unattended for long periods’. In Au et al.’s (2022: 7) paper, one respondent with Long Covid reflected, ‘you do not know frustration until you have had to advocate for your own care against a system that is reticent to adapt while you are debilitated by a novel illness that includes dense brain fog’. Extant literature illustrates the disabling nature of cognitive issues in PoTS and related conditions, both socially and in medical settings.

### Impacts of diagnosis

Receiving medical diagnoses such as PoTS may cause different emotions in different people and in Knoop and Dunwoody’s (2022: 1632-1633) study, one theme was ‘a mixed bag of emotions’, as some participants detailed challenges (such as feeling like a burden on others) but also gratitude (such as for the support from other people/partners). Monk (2024) and San Filippo (2020) described grieving their PoTS diagnoses with the latter explaining, ‘if I am being honest, I grieved. I grieved the woman I used to be and some days still do’ but that her mindset for this and other aspects of life is to ‘take it for what it is and learn to make the best of it’. However, in Frane’s (2023: 72) thesis, ‘out of all 12 participants, none described their final accurate diagnosis of POTS and/or EDS as a negative event’, despite ‘the conflicting feelings this may produce’, with the most cited emotion being ‘relief’. Kelly and Millward (2004: 6-7) stated that ‘the sick role paradigm established by Parsons (1951) […] is one of the most significant and important pieces of theorising about the social nature of illness of the last century’; This included ‘his idea that sickness was a form of social as well as biological deviance’ (Parsons, 1951). Cheshire et al. (2021: 299) stated that according to Parsons’ theory, the sick role ‘was entered into with a physician’s diagnosis’ and ‘entering this role was thought to free a person from some social expectations (e.g., work) and blame for being sick, while they temporarily occupied the role (Parsons, 1951)’. After I received my PoTS diagnosis, I felt somewhat freed from able-bodied expectations and have found it easier to accept myself as I am. I have noticed that, in some cases, my diagnosis has affected the way in which people treat me, as before I was asked sentences beginning, ‘are you sure you cannot.?’ and was told, ‘no, you’ll be fine to...’ but my limits are generally more socially acceptable now. When I reflect on the sick role and my own experiences, I feel that sometimes people only believe there is an excuse or reason for ill-health and altered functioning when a medical professional has agreed this. A person is still sick before they are diagnosed and I hope that in the future, there will be more societal acceptance towards the undiagnosed but unwell populations.

Cheshire et al. (2021: 300) stated that some theorists, ‘including Parsons himself, argue[d] that the theory highlights responsibility of the chronically ill person to minimise the effects of their health condition (rather than recover from it), by engaging with medical advice/treatment, displaying motivation to recover, and not “give in” to the illness (Varul, 2010)’; ‘Bury (1982) proposes that a chronically ill individual may only have periods where they occupy the sick role due to, for example, symptom flare-ups or surgery’ (Bury, 1982). In my experience, there can be a stigma attached to not being perceived to be displaying enough motivation or effort to recover and I have had time in and out of the sick role since puberty. Early on in my post-viral flare-up, several people who did not know about my situation confidently told me that they thought that people who claimed to have Long Covid were ‘faking it’, exaggerating or using it as an excuse to work less hard. I found this really awkward initially but have become more vocal over time as my symptoms have become more severe. I find this offensive and rude, as it implies that those suffering after the virus are not deserving of occupying the sick role and are to blame for their reduced or altered functionality. Long Covid is an example of a chronic health issue in which ‘feelings of invisibility and stigma due to others not understanding the condition are prevalent’ (Waterman et al., 2021: 189).

I feel that one of the main advantages of receiving my PoTS diagnosis was that I was able to begin accessing and taking medication. For the first 4 months after I began taking one of these, I had no episodes of conscious blackouts, fainting or drifting in and out of consciousness, compared to these occurring twice to three times per week on average before (since my flu jab). I had a pre-medication heart rate that jumped up to 150-180 bpm from minimal exertion but medication helps my heart rate to be less excitable now. However, one HCP has been vocal with me about feeling that I should come off the medications prescribed by the dysautonomia specialist (in favour of going on other medications suggested by him). He argued that conditions such as PoTS and ME/CFS are not based on proper science, that I should identify as having ‘a post-viral syndrome’ instead and that too many people with post-Covid symptoms are being falsely diagnosed as having syndromes such as PoTS and ME/CFS (which he stated that many HCPs do not ‘believe in’) when they are just deconditioned and need to exercise more. Because I have no medical background, I find the varied perspectives on using medication for PoTS and on the contested validity of these conditions stressful and confusing. I find that interactions with friends or others who have not experienced chronic illnesses themselves illustrate a naivety that receiving a diagnosis means that health challenges will end, almost automatically. In Knoop and Dunwoody’s (2022: 1633) study, ‘making sense of symptoms was very individualistic and a diagnosis did not always lead to instantly reliable management strategies’ for those with PoTS, as much like in ME/CFS and Multiple Sclerosis (MS), ‘there was an emphasis on the need to “get to know” their own symptoms, some of which were difficult to control’. I have experienced—and continue to experience—very conflicting advice on managing my dysautonomia. I believe that regardless of an HCP’s perspective on diagnostic labels, they should show compassion for the patients experiencing challenging symptoms. In my post-viral experiences, those who reject labels such as PoTS, ME/CFS and Long Covid tend to also invalidate my symptomatic experiences, which I find more hurtful and unhelpful than the questioning of the labels themselves.

PoTS UK (b) explained that there are no approved medicines for treating PoTS, thus medications are prescribed ‘off licence’. ‘Treatments must be tailored to each patient, taking into account the cause of their PoTS’, individual symptoms, co-existing conditions and side effects (PoTS UK, b), as well as any interactions with other medications. Arotin (2019) described taking ‘medication to lower my heart rate, antinausea tablets, dizziness tablets, and car sickness medication, all in an attempt to reduce my symptoms’ before seeking alternative therapies and Sara, a patient from the PoTS Treatment Center (n.d.), explained that she was ‘just covering up [her] symptoms with medications’ so she ‘needed a true solution’. I find that those who have not experienced chronic illnesses themselves often wrongly assume that doctors always choose which medications their patients take and that this is a quick decision, rather than a more personal process. Some people do not want to take medication, while it may prove ineffective for others and may or may not be worth the side effects or potential long-term risks. On PoTS UK (a), James explained that medication really helped him but Kira stated that the same medication made her symptoms more severe. Conor described having PoTS and other conditions and stated, ‘medication unfortunately does not work with my body and has little effect on my symptoms’ (PoTS UK, a). While two medications that my dysautonomia specialist prescribed have been brilliant, I could not withstand the side effects of a third and came off it after only a few weeks. I experienced challenging side effects from all three initially, as like San Filippo (2020) explained, ‘with POTS, my body is very sensitive to medication’. PoTS UK (c) described four ‘causes/subtypes’ of PoTS: Neuropathic, Hypovolaemic, Hyperadrenergic and Deconditioning. Different causes and types can affect the suitability of medications and nonpharmacological interventions so it should not be oversimplified that all PoTSies improve with more salt, medication or other changes. Some friends/family have excitedly told me about people with PoTS who have tried something and gotten much better (or recovered entirely), however, their advice has not appeared to work for me so PoTS interventions can have varied success for different individuals.

O'Connor (2024: 2) has Long Covid and ‘meets diagnostic criteria’ for PoTS, MCAS and ME/CFS, as well as showing ‘signs of immune system dysfunction’. She explained that ‘Long Covid is a debilitating and devastating chronic illness’ causing ‘loss of employment, inability to parent your children, breakdown of marriages, and destruction of your identity and life as you previously knew it’ (O'Connor, 2024: 9). This links to Bury’s (1982) concept of ‘biographical disruption’, which is ‘the influence of a significant, sudden event or events on the course of an individual’s life that cardinally changes its direction and plans’ (in Pranka, 2018: 1). Illnesses can break ‘an individual’s social and cultural experience by threatening [their] self-identity’ (Pranka, 2018). I have experienced biographical disruption due to my severe COVID-19 symptoms continuing to have debilitating impacts on me over 2 years after my initial infection. Stopping to reassess my life while spending great lengths of time in bed with little ability to tolerate distractions (such as music or TV shows due to exhaustion and noise/light sensitivity, as well as involuntary sobriety due to alcohol-induced flare-ups) helped me to realise things about my life that I wanted to change and ways in which I wanted to better distribute my energy in future. It caused me to come out to my friends and family about being bisexual and believing I may be autistic, as well as encouraging me to live and communicate more authentically in general. This could be conceptualised as fitting into Frank’s (1995) quest narrative in which there are unexpected positive effects from illness (Spillmann et al., 2017: 1–2). When I first got unwell from COVID-19, my friend called it ‘divine intervention’ due to its significant upheaval on my life and her feeling that I needed something like that to happen. My perspective on this fluctuates, as often I think it may be true but when I flare-up, for example having more than 20 migraines in a month, I do not, as I utilise more of a chaos narrative of suffering and loss (Spillmann et al., 2017). My poem in Figure 1 illustrates some of my conflicting emotions about my post-Covid diagnoses’ impacts on my life.

### Recovery and expectations

PoTS can cause a variety of symptoms and variable outcomes (Pandian et al., 2007: 529). There are divisive academic arguments and public discourses about the extent to which remission and/or recovery are possible for everyone with PoTS, with Knoop and Dunwoody (2022: 1632) stating, ‘currently, there is no cure, approved or licenced treatment for POTS’ but that individuals create self-management strategies. Kizilbash et al. (2014: 1), who explored adolescents’ experiences of PoTS, argued that ‘full recovery is possible with multi-faceted treatment’ and ‘aerobic exercise is a key to successful recovery’ (Kizilbash et al., 2014: 29). They asserted, ‘patients with POTS should resume regular physical and academic activities. Sometimes, this must be done in an incrementally increasing fashion over several weeks, but recovery from POTS hinges on avoidance of daytime recumbency and inactivity’ (Kizilbash et al., 2014: 19). In Knoop and Dunwoody’s (2022: 1633) study, no participants expected to be ‘cured or symptom-free’ but one participant hoped for day-to-day improvements, ‘advocating for better management and, ideally a cure being discovered someday’. However, another participant ‘felt that a cure was a long way off and of little consequence to her current situation’ and her ‘resigned outlook’ was echoed by another participant who ‘despite having been told that POTS may possibly get better over time, [...] held a fatalistic attitude and limited hope of ever fully recovering, coupled with a prevailing sense of uncertainty about how her symptoms may worsen’ (Knoop and Dunwoody, 2022). San Filippo (2020) described her PoTS as ‘a chronic illness. Chronic meaning it will be around for, well… ever, or at least until some genius finds a cure. At first, it took me a while to swallow that sentence. I will forever have to deal with this illness. My life was and is forever changed by this diagnosis’.

PoTS ‘can be mild to disabling’ with most PoTSies experiencing fluctuating pain and symptom severity (The Dysautonomia Project, 2015). Some dysautonomia patients become bedridden due to their illness (Dysautonomia International, n.d.) and The Dysautonomia Project (2015) asserted that ‘approximately 25% of POTS patients experience symptoms so severe that they are unable to attend school, work, drive, and some are bedridden’. I have met people who have had to quit their jobs/careers due to their Long Covid and subsequent PoTS and fatigue (thus, entering into the sick role) but have also met people who feel their PoTS barely affects them, as it is ‘under control’ or simply impacts dizziness upon standing and little else. I was surprised (and inspired) to read of some PoTSies who have accomplished exercise-related goals such as being able to run races. Cathi, a PoTSie, ‘runs 5 k races while pushing a wheelchair, in case she feels too dizzy’ (Lisa, 2014) and Tulley (n.d.) detailed her challenges, accommodations and accomplishments in running ultramarathons with PoTS. Tulley (n.d.) stated, ‘It is my dream to complete 100 miles, and it is a bigger dream to beat POTS, be a fast runner again, and win a 100-mile race. [...] I know I can do it, I know there is a formula, and somewhere there is a cure for POTS waiting to be discovered’ before later adding, ‘I still have POTS, but it is not dictating my life’. In Jade’s story on PoTS UK (a), she stated that it is confusing for her and those around her that on some days, she can ‘go for a run and be what looks like normal (the truth is I mask my symptoms) but then there are days that I can get up and faint or I’m completely unable to get out of bed at all’. Thus, not only can PoTS vary between different individuals but it can also vary from 1 day to the next for each person.

In a quest to uncover whether she could run a marathon with PoTS, Monk (2024) explained, ‘I wasn’t searching for a cure. I was just desperate to find out if more was possible in my life’. Detailing taking many walking breaks and running with friends who slowed down for her, she reflected, ‘when we stopped to walk, we showed each other that needing a break—needing help—does not have to mean getting left behind. My nervous system still sucks, but that simple solidarity healed something deeper in me. Running a marathon did not take away the grief that came with my POTS diagnosis. But it gave me a way to practise letting others help me through hard things’ (Monk, 2024). This shows that interactions at the level of the individual, including conversations with friends, can impact PoTSies’ abilities to participate in activities, set and meet personal goals and cultivate a happier QoL. In my post-viral experience, it is difficult to entertain the idea of gradually increasing my exercise to run races as the more I do, the longer I seem to suffer for. My attempts at slightly improving my standing and walking times have not resulted in improvements to my mobility, instead giving me more frequent migraines and worsened fatigue symptoms. While my first draft of this manuscript has been in the review process, I received a diagnosis of ME/CFS so I am in the preliminary stages of understanding what this means to me emotionally and physically, how it impacts my exercise (in)tolerance and how it affects my recovery prospects. While I do not relate to training to run a marathon and currently need to sit down to fry an egg, the idea of adapting a task to make it possible and surrounding myself with people who understand how I might need to do things differently resonates with me, with the extent to which I can participate in activities significantly affecting my wellbeing. I find reading too many stories of particularly mild or severe PoTS, including narratives of immense improvement or deterioration, unproductive for me, as I find it most helpful to focus on my own body, boundaries, goals and dreams (which is easier said than done sometimes).

When Bryarly et al. (2019: 1225) discussed POTSies becoming ‘more active and mobile as they embark upon their path to recovery’, this made me think about Cheshire et al.’s (2021: 301) research on what ‘recovery’ means to people with ME/CFS, as they found that ‘the meaning of recovery differed between participants’. This is interesting, partially because of the similarities between PoTS and ME/CFS but also because ‘chronic fatigue has been cited in up to 48% of POTS patients’ and ‘patients with CFS often have POTS as well’ (Bryarly et al., 2019: 1216). ‘In medical terms, this could be considered a return of one’s health to that before illness’ with some participants appearing to define recovery as being ‘100%’ symptom free or ‘able to do everything other healthy people could do’ (Cheshire et al., 2021: 301) but others ‘appeared to respond by moving their “recovery goal posts”—highlighting the achievement of obtainable goals over a full return to health’ (Cheshire et al., 2021: 302). This included one participant saying that she wanted to enjoy ‘personally rewarding and meaningful activities, such as creative endeavours and being able to have fun’, stating ‘even if I do not recover completely but if I got to a stage where I could do some fun things’ such as having a social life, not being housebound and travelling home to see her family (Cheshire et al., 2021: 303) as what she hoped for. When reflecting on what recovery means to me, I asked my 14-year-old sibling for help and the response I got was, ‘for your PoTS, recovery would be more Bro Sis time [the time we spend together], more work, more going out with friends for nice food and more fun’. I loved this answer and the idea from some of Cheshire et al.’s (2021) participants that it does not have to mean a full return to previous health which I do not feel I have control over but instead offers more focus on joy, which I am able to work towards.

This idea of recovery and adjusting expectations reminds me of two friends who frequently used the phrase ‘when you are better’ to me and how this can feel. When reflecting on interactions at the level of the individual, I have remembered the kindness, thoughtfulness and empowerment that I have received from my five closest friends and new postgraduate friends during my post-virus journey with Long Covid and PoTS, for which I am very grateful. It has also reminded me, however, of a few comments from my wider circle of friends that I have found to be quite irritating or dismissive. When having to decline social offers due to ill-health, there have been times when I have explained my limitations, offered alternative plans and hoped to still be able to keep in contact with others but there has been a lack of interest in return to go to cafés, call, meet locally, socialise for shorter lengths of time or meet without alcohol (the latter being important to me due to alcohol’s exasperation of OI; Bryarly et al., 2019: 1222–1,223). Kelly and Millward (2004: 5) asserted that ‘illness states [can] have consequences for self and identity’ and ‘identity changes’ was a key theme in Waterman et al.’s (2021: 191) study, as ‘overall participants reported a significant impact to their identity and sense of self since developing PoTS’. The disinterest to scale down or alter plans makes me feel like I am not perceived as being worth seeing or contacting and that I am not enough as I am now; To me, it suggests that only my past self and a potential future version of me (that may or may not ever exist) qualify as deserving of social interaction, company and inclusion and that I am perceived in terms of what I can offer and do, not in terms of who I am.

‘A frequently described comorbidity in POTS patients is migraine, as well as other chronic headache types’ (Bryarly et al., 2019: 1216); ‘Intractable migraines often lead to physical inactivity, which may exacerbate orthostatic intolerance, and conversely, the increased sympathetic activity associated with POTS may contribute to increased frequency of headaches’ (Bryarly et al., 2019). After a week of one of my worst post-PoTS-diagnosis migraines, a little boy who looked about 6 years old shouted ‘excuse me’ and asked me where I was walking so I replied that I was going to the end of the road and smiled before turning to keep going. ‘Dats [meaning that’s] NOT far’, he said as he furrowed his brow. I laughed to myself that he was a physical manifestation of my self-critical inner monologue and replied, ‘I’m happy with that for today’. He carried on, ‘but dats not far! It’s only over there! Dats not far’. I shrugged, smiled and kept going. When I spoke to my uncle about the improvements I have experienced socially, physically and emotionally since beginning to understand OI and dysautonomia, I nonchalantly mentioned that my pre-diagnosis experiences were ‘inconvenient’. He replied, ‘no, at its worst, it’s been terrifying’. Before I had an MRI on my head and ears, my migraines frightened me, as did my syncope before I understood its causes. I may not be ‘recovered’ in the sense that I have a disability which is still disabling (medically and socially) but I am recovering from being in a period characterised by significant fear and uncertainty. I hope to have conveyed some of these emotions in the poem that I have written (see Figure 2).

### Community

In Rich et al.’s (2020: 9) study, ‘participants held mixed feelings on the benefit of support groups, with some feeling better understood and others feeling triggered or brought down’. Laird-Gion et al. (2022: 211) ‘completed a feasibility study of a virtual, interactive, empowerment-based small-group workshop for patients with POTS’, which was ‘created and presented by […] physicians and patients’. The session topics were ‘Introduction to POTS’; ‘Nutrition, Salt, and Compression’, ‘Exercise’ and ‘Living with POTS’ (which included ‘how to talk about illness with friends and family’; Laird-Gion et al., 2022). Although living with PoTS can be challenging, chronic illness communities, workshops and support groups can be helpful for some PoTSies. In Knoop and Dunwoody’s (2022: 1632) study, most PoTSie participants had ‘made use of online support groups’ in an ‘attempt gain self-management information’, ‘however, these tended to reinforce negativity’ with one PoTSie saying, ‘it does not feel productive for me’. Similarly, in my experience, Facebook groups for PoTS and other chronic illnesses can feel overly negative and unproductive, however, I found Tik Tok to be an accepting, humorous and helpful community for learning about PoTS and feeling empowered to manage it. While content on the platform can be criticised for encouraging self-diagnosis of PoTS, interactions that I had with PoTSie Tik Tokers felt productive and offered me a sense of belonging in contrast to the isolation that can come with being unwell, losing friendships and socialising less. In Frane’s (2023: 66) study, a participant with EDS who was awaiting a potential PoTS diagnosis explained, ‘because I’ve gone my whole life without a diagnosis, I’ve worked out that I need to pace myself with contacting medical professionals in terms of the admin and the emotional trauma of just simply trying to get medical help. It is so hard.’ She noticed in PoTS and EDS communities, ‘a large prevalence of people with these illnesses who also suffer with poor mental health, perhaps due to the experiences of medical gaslighting that many have to contend with’ (Frane, 2023). Seeking advice from support groups, virtual communities and platforms such as Tik Tok may be perceived as a welcome alternative to the risk of being invalidated in medical settings.

One of the main positives of chronic illness communities, for me, is the freedom of discussing chronic illness without concerns of how to present my symptoms and experiences in a way that is comfortable for able-bodied people without the fear of stigma. Tik Tok can form an accessible way to learn about the condition without having to read too much (which can be useful for those with fatigue and brain fog). When reflecting on managing her ulcerative colitis socially, Moore (2013: 204) stated, ‘I lean against the wire fence and try to hide my hunch of pain by pretending to undo my shoelaces’. When she was asked ‘mate what’s up?’, she replied, ‘my stomach. It’s just being really weird at the moment, hurts when I run’ (Moore, 2013). Because loud sounds can trigger my PoTS, I often wear noise-reducing earbuds to prevent flare-ups during loud group conversations and when in busy public spaces. This idea of playing down pain or trying to manage it subtly resonated with me as when choosing my earbuds, I chose subtle colours so that they would hopefully not draw much attention and when asked about them, I rarely disclose the sheer pain and discomfort that I may experience from sensory sensitivities, tinnitus and a migraine if I do not wear them. In Rich et al.’s (2020: 5) study, ‘sensitivities to smells, temperatures, sounds, and lights impacted socialization, childcare, bathing, attendance at religious gatherings, and grocery shopping’. I find that most people who do not experience sensitive sensitivity or sensory-induced symptoms struggle to comprehend how challenging they can be or how much thought goes into managing them. Moore (2013: 204) reflected, ‘my pre planned line, akin to “covering,” (Goffman, 1963) offers little to her but in my mind it is enough’ and explained that Goffman (1963) argued that people ‘who are ready to admit possession of a stigma (in many cases because it is known about or immediately apparent) may nonetheless make a great effort to keep the stigma from looming large’; ‘The individual’s object is to reduce tension, that is, to make it easier for himself and the others to withdraw covert attention from the stigma’ (Goffman, 1963).

Moore (2013: 204) stated, ‘that’s as much as I give’, as she does not say ‘every time it hurts, I have to go to the loo and it takes all my energy not to be reduced to tears’ or ‘I’m struggling to cope with getting things done’. I relate to this disparity between the challenging reality and the version of events presented to others. In Goffman’s (1959: 9) ‘The Presentation of Self in Everyday Life’, he considered ‘the way in which the individual in ordinary work situations presents himself and his activity to others, the ways in which he guides and controls the impression they form of him, and the kinds of things he may and may not do while sustaining his performance before them’. I tend to find that when speaking to fellow chronically ill people, whether they have PoTS or very different conditions, I feel less concerned about Goffman’s (1959) idea of impression management or spoiled identity (Goffman, 1963) and more comfortable to be honest and vulnerable about my situation. I attend group hypnotherapy for people with Long Covid, ME/CFS and/or PoTS and in these sessions, the sense of belonging and shared understanding negates the need to mask or obscure challenges, pain and limitations. I find this more productive than interactions on Facebook support groups because we all attend due to wanting to feel better, calmer and more empowered through the hypnotherapy so we have a shared focus and desire to improve and/or accept our situations. When talking to fellow chronically ill people, I value being able to be unfiltered and not having to worry about whether my reality would be stressful, depressing, repetitive or boring for them to hear.

## Discussion and conclusion

This qualitative, sociological article explored the invalidation that can arise from trying to receive and manage a diagnosis for an often-invisible, understudied health challenge. Difficulties with misdiagnosis, communicating and declining plans were discussed, as well as the varied experiences of medication. Auto/biography ‘disputes the conventional genre distinction between biography and autobiography, as well as the divisions between self/other’ and ‘public/private’ (Stanley, 1993: 42). Having struggled with presyncope and symptoms conceivably caused by dysautonomia for over a decade, I reflected auto/biographically on the decision to use a mobility aid and to label myself as ‘disabled’, as well as my interactions in and out of occupying the sick role. Depending on others, adjusting plans and reconsidering what ‘recovery’ means to me can help me to cope with the uncertainty of having PoTS and Long Covid, while both nonpharmacological and pharmacological interventions (such as increasing salt and taking medication) have helped me practically with symptom management/predictability. Pre-diagnostic interactions can involve disbelief, uncertainty, fear and frustration for PoTSies, while interactions with chronic illness communities can (with varied perceived success) offer spaces with less pressure to engage in impression management to avoid stigma.

While my initial research aims were to understand the impacts of social interaction, condition invisibility and misdiagnosis/delayed diagnosis on individuals with PoTS, additional themes and sub-themes emerged during conduction of my review such as community, running, wheelchair use, varied experiences with medication and expectations for the future. Partially because of the lack of relevant qualitative research specifically about PoTS lived experiences, I also drew from non-academic sources such as blogs which detail experiences and perspectives on managing PoTS (such as Lisa, 2014), as well as academic evidence from wider qualitative lived experience literature (such as Joachim and Acorn, 2001). This article covers a variety of perspectives, arguments and experiences, including those that differ from my own. For example, I struggle persistently with mobility issues and intermittently with cognitive issues, whereas others diagnosed with PoTS may experience the opposite. Causes/triggers of PoTS can vary, for example, including sepsis for Jade, COVID-19 for Ellie and Chloe, tonsillitis for Sophie and Glandular Fever (EBV) for James in their (PoTS UK, a) health stories. For others such as Conor and Rebecca, symptoms may be present before notable events but then become worse, for example, Rebecca’s symptoms came and went until she experienced flare-ups due to having her appendix removed and later being pregnant. Some people with PoTS feel they will or will not recover fully, whereas I feel uncertain. Future research should explore the extent to which varied beliefs about recovery are affected by other factors such as the causes of developing PoTS or length of time spent undiagnosed. Symptoms and severity of PoTS vary, as well as lived experiences and their impacts on identity and wellbeing.

The term ‘POTS’ was first used in 1993 by Schondorf and Low from Mayo Clinic, ‘however, reports about similar conditions can be found earlier in the medical literature’ (Fedorowski, 2019: 352). Because the term ‘POTS’ (which can also be written as ‘PoTS’) was first used only 30 years ago, research, understanding and awareness of this chronic illness are in their infancy. While the increased experiences of PoTS and PoTS-like symptoms due to the COVID-19 pandemic are very unfortunate and challenging, it is hopeful that the increased awareness of the condition will help to improve understanding from HCPs and wider society. A limitation of this study is that understanding of Long Covid is contentious and the prospects are yet to be fully known or understood. My paper focused on interactions, rather than on other impacts of experiencing PoTS such as financial challenges (Rich et al., 2020: 7) due to what is known as the disability price tag. A theme in Knoop and Dunwoody’s (2022: 1633) study was titled, ‘I’m expensive in so many ways’, which included issues socially, emotionally and financially. Participants discussed the expense of healthcare in the US and issues such as loss of earnings (Knoop and Dunwoody, 2022), which future research should address in more detail. My research can only be based on what is known so far about each of the conditions and experiences discussed without foresight about the future research and understandings that may emerge. PoTS is the most prevalent form of OI and dysautonomia but future research should explore the lived experiences of individuals with other forms of autonomic dysfunction and OI too, for example, the thoughts, feelings and experiences of those with IST, which is ‘sometimes confused with POTS, but occurs independent of body position’ (Bryarly et al., 2019: 1215). Future studies should also seek to understand the positive and negative impacts of new, emerging virtual chronic illness communities such as those on TikTok and the motivations leading PoTSies to get their information from there.

More research should explore health issues predominantly affecting women/AFABs, as it is argued that these have not received enough research focus thus far. The impacts of expectations of masculinity on men/AMABs with PoTS should also be explored due to the findings about those with SCD (Poku and Pilnick, 2022). A limitation of my paper is the lack of focus on associated conditions so future research is needed to explore the experiences of those with dysautonomia and its potential comorbidities. ‘Reported comorbidities in children with POTS mainly include allergic disorders, migraine, psychological disorders, hyperventilation syndrome, chronic fatigue syndrome, hypermobile Ehlers-Danlos syndrome and hypermobility spectrum disorder (hEDS/HSD), gastrointestinal dysfunction, and fibromyalgia’ (Wang et al., 2021: 8946). Current understandings of comorbidities of PoTS tend to vary with some links seeming likely but not well understood. For example, ‘despite well-established clinical associations’ between hEDS and PoTS, ‘the precise prevalence is unknown’ (Miller et al., 2020: 1) and MCAS is ‘a relatively unknown condition that may affect some people’ with PoTS, however, ‘PoTS and MCAS are not very well understood and the overlap between the two conditions is complicated’ (Clarke and Nicholson, 2021). ‘Observational studies indicate that up to 30–40% of convalescent COVID-19 patients develop chronic widespread pain and fatigue’ and fulfil ‘the 2016 diagnostic criteria for “fibromyalgia.”’ (Plaut, 2023: 1) and ‘“Long COVID-19″ exhibits fibromyalgia-like manifestations and symptomatology including chronic fatigue, cognitive impairment, low mood, functional impairment, and last but not least-myofascial pain (i.e., “fibromyalgia-ness”)’ (Plaut, 2023: 10). Future research is needed to understand the potential connections between PoTS and conditions such as hEDS, MCAS, and fibromyalgia and the lived experiences of those diagnosed with a combination, including that of individuals who have developed PoTS as a secondary condition (meaning, their PoTS is caused by a different condition).

There are also limitations in terms of my methodology. As I conducted qualitative research, my findings are not as generalisable, reliable or representative as that of quantitative studies such as large-scale surveys on how PoTS presents for a wide variety of people. These issues are also exacerbated by the lack of research on the lived experiences of individuals with PoTS, as there was a paucity of relevant qualitative, academic studies for me to draw from and analyse. Frye et al. (2022: 623) stated, ‘the current literature review [meaning their paper’s review of past research] did not identify a single study using qualitative methodology to explore the experiences of patients living with POTS’. While, thankfully, there are some papers like this now, there are simply not enough. Although I referred to the first 10 patient stories visible on PoTS UK (a), future research should seek to represent and analyse more, if not all, of these, as their lived experiences are insightful. Knoop and Dunwoody (2022: 1634) stated under their ‘limitations’ subheading that ‘the lead researcher had a diagnosis of POTS, so there was a risk of researcher bias’ in their paper, which was countered using a reflexive diary with results audited by a second researcher. While I believe this was a good decision and that avoiding researcher bias when possible is important to ensure academic rigour and robustness, I also feel that sharing researchers’ positionality and lived experiences can be valuable particularly in such an under-researched area. However, illustrating the often-dynamic nature of chronic illnesses, I was struck by how much has changed in my health and my conceptualisations of what I have experienced in the relatively short time between submitting my first draft manuscript and updating it according to revisions. For example, I was infected with COVID-19 for a third time (causing a severe, debilitating flare-up) and have been diagnosed with ME/CFS. I am still in the preliminary stages of understanding what these changes mean to me emotionally and physically so my paper primarily remained focused on my lived experiences before these events. It has proved difficult trying to decide the extent to which to adapt my paper in some ways to reflect changes in my positionality versus the desire to ensure it remains representative of how I felt when I first wrote it. Regardless of this methodological difficulty of writing auto/biographically, I ultimately hope that sharing my story will make a difference, even if just to one person.

From a chronically ill patient’s perspective, I recommend that HCPs are more validating towards patients with unexplained symptoms and/or PoTS in future, as interactions with HCPs can greatly impact our lived experiences, wellbeing and perceptions of ourselves. It is crucial to avoid and challenge any potential discriminations due to sexism, ageism, disablism and ableism. I hope that my poetry and lived experiences can help to improve future interactions for those with PoTS due to increased public awareness and compassion. I recommend that anyone who wants to learn more about the lived experiences of those with PoTS reads some of the patient stories on PoTS UK (a), as these are accessible and powerful, illustrating the diversity of experiences of those with the condition. When considering my experiences, I am very grateful for the medical tests that I had access to thanks to my primary HCP and the NHS, as well as some of the positive and validating interactions that I have experienced. However, I remain ‘salty’ about the challenges faced by those who are attempting to uncover the causes of their symptoms including for PoTSies who are dismissed and ignored. I am also ‘salty’ about the lack of public understanding towards those with invisible illnesses who feel obliged to try to disguise their challenges or are delegitimized when symptom severity varies over time. I encourage researchers in the medical and sociological fields to research PoTS so that understanding of how to treat it, and treat those who have it, can improve.

## Data availability statement

The original contributions presented in the study are included in the article/supplementary material, further inquiries can be directed to the corresponding author.

## Ethics statement

Ethical approval was not required for the study involving humans in accordance with the local legislation and institutional requirements. Written informed consent to participate in this study was not required from the participants or the participants’ legal guardians/next of kin in accordance with the national legislation and the institutional requirements.

## Author contributions

HM: Writing – original draft.
